# Strategies for and Barriers to Managing Weight When Eating at Restaurants

**Published:** 2010-04-15

**Authors:** Gayle M. Timmerman, Marie Earvolino-Ramirez

**Affiliations:** University of Texas at Austin; University of Texas at Austin, Austin, Texas

## Abstract

**Introduction:**

Eating in restaurants contributes to excess caloric intake, which leads to weight gain, but little is known about strategies used to manage weight or barriers to weight management in restaurant settings. We describe and compare the strategies men and women use and the barriers they encounter when eating at restaurants.

**Methods:**

We recruited a convenience sample of 146 adults at a university open house. Participants completed questionnaires on demographics and eating patterns, strategies used to manage weight in restaurants, and barriers to managing weight in restaurants.

**Results:**

The most common strategies used by participants were avoiding sugar-filled drinks, choosing steamed vegetables and whole-grain foods, and stopping eating when full. We found few differences by sex: women were more likely to share appetizers or meals, substitute appetizers for meals, have salads as entrées, order salad dressing on the side, and bring half of the meal home.

**Conclusion:**

Women and men had more similarities than differences in strategies for and barriers to managing weight in restaurants. We need to understand what influences food choices at restaurants in order to develop comprehensive plans for weight management.

## Introduction

Obesity increases a person's risk for developing chronic health problems such as hypertension, diabetes, cardiovascular disease, and stroke (1). Because most US adults are overweight or obese (2), weight management is essential for chronic disease prevention. Dramatic increases in the prevalence of obesity in the past 25 years are attributed to an environment that promotes excessive calorie intake, coupled with a sedentary lifestyle (3). Eating in restaurants contributes to this excess intake; Americans are 40% more likely to eat out at least 3 times per week now than they were in the 1980s (4), often eating large portions of calorie-dense foods (3).

Frequency of restaurant eating is positively associated with calorie and fat intake, along with body fat (3-6). Other studies show positive associations between frequency of eating fast food and body mass index (7-8). Little is known, however, about how restaurant eating affects weight management. We conducted a Medline search and found no studies that specifically addressed strategies used to manage weight or barriers to weight management when eating out. Studies have found that several factors influence restaurant eating behavior: taste, portion size, emotional needs, perceived value, and social interaction (9-17). These factors are often barriers to weight management when eating out.

The main objective of this study was to describe how often women and men use different strategies for managing weight when eating out (including fast food) and the magnitude of barriers to managing weight when eating out. Because sex differences occur in food choices and weight management behaviors (18-20), a secondary objective was to explore sex differences in the specific strategies used and the barriers encountered to increase our understanding of weight management in restaurant settings.

## Methods

This descriptive survey study used a convenience sample recruited in the spring of 2006 during a campus-wide open house at the University of Texas at Austin, so the adults sampled for this study consisted primarily of parents of children of all ages (preschool to high school). The study was approved by the university's institutional review board, and all participants were provided with a cover letter that described the study.

The only inclusion criterion for the study was being at least 18 years old. To capture a variety of eating strategies used in restaurants, we included participants who were not dieting because many people who use strategies to manage weight may not be actively dieting. Adults who passed the display table for the study were asked to participate. Participants completed 3 questionnaires: background information, strategies for managing weight when eating at restaurants, and barriers to managing weight when eating at restaurants. A total of 156 adults completed the surveys. Participants received a pencil for participating.

We do not know the rate of nonresponse; we could not track people who declined to participate. We excluded participants only if 10% or more items were missing on either the strategies or barriers questionnaires, which resulted in a final sample of 146 participants. According to power analysis with an α of .05 for a 2-tailed test and a power of .80, a sample size of 146 would be adequate to detect a significant difference in an independent *t* test if a medium-to-large effect size was present (Cohen *d* > 0.48).

### Instruments

The background information survey collected information on age, sex, race/ethnicity, educational level, and eating patterns. The questions about eating were as follows: 1) “How often did you eat out (including fast food) in the past week?” 2) “How often do you try to manage your weight by watching what you eat (rarely, occasionally, sometimes, often, usually)?” 3) “Do you tend to eat in response to emotions (yes or no)?” and 4) “How many times have you lost 20 pounds or more and gained back at least half of it?”

The strategies questionnaire had 30 items that rated how often participants used weight management strategies while eating out. The Likert-type scale responses included "rarely (≤20% of the time)," "occasionally (21%-40% of the time)," "sometimes (41%-60% of the time)," "often (61%-80% of the time)," and "usually (81%-100% of the time)."

The barriers questionnaire had 25 items that measured the magnitude of barriers to weight management when eating out. The 5 possible responses were "not a barrier," "a small barrier," "a moderate barrier," "a major barrier," and "an overwhelming barrier."

Both instruments contained items that pertained only to full-service restaurants or fast food restaurants and items that pertained to both. We developed both instruments on the basis of literature review, previous research, and participant feedback from earlier research (6,11-14,21). For content validity, instruments were reviewed by a panel of 5 experts who rated the relevance and clarity of each item on a 1 to 4 scale (1 was lowest and 4 was highest). Most experts rated each item 3 or 4 for both clarity and relevance. Several items were reworded and simplified on the basis of expert panel feedback.

We tested the revised instruments for face validity on a convenience sample of adults (N = 56). The Cronbach α was 0.88 for the strategies questionnaire and 0.92 for the barriers questionnaire. On the basis of participant feedback, we rearranged the order of items in the strategies questionnaire to move the 3 alcohol-related questions to the end of the survey to be completed only if participants drink alcohol.

Although neither instrument was designed to have a subscale structure, we conducted factor analysis for each instrument separately. With principal axis factoring, the strategies instrument yielded a 2-factor structure; the 3 alcohol-related items loaded together (>0.7) on the second factor, and 24 of the remaining 27 items loaded on the first factor (>0.3). Because we designed items from the strategies instrument to describe individual strategies used and because only alcohol-related strategies loaded together, all items were retained so that we could examine results of individual items. Additionally, the barriers instrument did not yield any underlying factors. Therefore, we retained all items and examined individual items.

For this study, the Cronbach α was 0.90 for the strategies instrument and 0.91 for the barriers instrument. Item analysis showed that interitem correlations for all items on both instruments were from 0.30 through 0.70, which indicated that redundancy and relatedness to the concept were not a problem. We also analyzed item-total correlations. An item-total correlation more than 0.20 suggests that the item is substantially related to the concept being measured (22). Two of the item-total correlations for the strategies instrument were less than 0.20, but the items were not eliminated because of their established importance in the literature and participant feedback. The item-total correlations for the barriers instrument were all more than 0.20.

The summed score for the strategies instrument was significantly related to how often participants reported watching what they ate (*r* = 0.46, *P* < .001), which showed concurrent validity since people who more frequently diet would be more likely to use weight management strategies at restaurants. Summed scores for the strategies and barriers instruments were not correlated, which indicated that these 2 instruments measured 2 separate phenomena.

### Statistical analysis

We used SPSSX version 15.0 (SPSS, Inc, Chicago, Illinois) to calculate descriptive statistics. To summarize strategies that were used the most, we combined and ranked frequencies for the categories of "often" and "usually." To summarize what the participants perceived as major barriers, we combined and ranked frequencies for the categories of "major" and "overwhelming." To explore what participants perceived as not being problematic to managing their weight when eating out, we combined and ranked frequencies for the categories of "not a barrier" or "small barrier."

We examined sex differences for the mean scores of individual items on both instruments by using *t* tests and the Mann-Whitney U nonparametric test. We considered differences to be significant at *P* < .05. Although Bonferroni adjustments are often used when multiple comparisons are made to control the family-wise error rate (type I errors), these adjustments increase the likelihood of making type II errors, in which differences are missed. Some experts recommend discussing the potential problems and letting readers draw their own conclusions without the use of Bonferroni adjustments (23). Since this is a new area of inquiry, we did not use a Bonferroni adjustment, and we discuss the increased possibility of a type I error under "Limitations."

## Results

Of the 146 participants who completed the study, 56% were non-Hispanic white, 30% were Hispanic/Latino, and 3% were African American. The mean age of the sample was 38 (standard deviation [SD], 11) years, and 62% were women. Most of the sample was well educated (84% had at least some college).

Participants ate out an average of 3.5 (SD, 2.8) times per week, and men and women did not differ in frequency of eating out. Of the 41% of participants who reported eating in response to emotions, most (70%) were women. More than half (60%) of the participants reported that they watched what they ate to manage weight at least sometimes. Weight cycling occurred an average of 1 (SD, 1.5) time. We found no significant differences between men and women for educational level, ethnicity, frequency of managing weight by watching what you eat, eating in response to emotions, or number of times weight cycling occurred.

The top strategies for managing weight for both women and men were to avoid sugar-filled drinks, choose steamed vegetables, stop eating when full, and choose foods made with whole grains (Table 1). Strategies that were rarely used for both women and men were to have the bread or chip basket removed from the table, have a low-calorie snack before going out to eat, and ask the chef to prepare a menu item in a low-calorie or low-fat manner.

Of the 30 strategies, use of 6 differed significantly by sex (Table 1). Compared with men, women more frequently shared appetizers, substituted an appetizer for a meal, ate a salad for the main course, ordered salad dressing on the side, had half of the meal packaged to go, and shared a meal with a dining partner. All differences that we found to be significant with a *t* test retained significance when we used the Mann-Whitney U test, a nonparametric test.

The leading barriers for both women and men were that a busy lifestyle results in being overly hungry when eating out, restaurant food tastes good, and not wanting to waste food (Table 2). Factors that were not barriers were finding it hard to ask wait staff to package meal to take home, not wanting to take leftovers home, difficulty getting others to share an entrée or dessert, wanting to eat the same thing as dining companions, and eating out when not hungry to please others. The only barrier that was significantly different between men and women was difficulty in having the bread or chip basket removed from the table: women were more likely than men to perceive it to be a barrier.

## Discussion

Participants varied in how often they used different weight management strategies; numerous strategies were used regularly by some but not by others, which indicates that strategies for managing weight in restaurants should be individualized. Further research on factors that influence food choice and weight management behaviors in restaurant settings is needed to clarify how weight management can best be incorporated into restaurant eating. Additional research is also needed to establish the effectiveness of preventing weight gain when specific strategies for weight management in restaurants are used.

Women and men in this study had few significant differences in strategies used to manage weight in restaurants or in the barriers they encountered. Of the 6 differences in strategies, 5 involved reduced portion size and 1 involved eating salad as an entrée. One possible explanation is that American cultural standards include men eating heartily (24). Some people may view a hearty appetite and red meat consumption as masculine (25,26). Since eating out often occurs in social settings, some men may eat large portions of food or refuse to eat a salad as a main course so that they will be viewed as more masculine. Further study of sex differences in weight management strategies is needed with larger samples to validate the findings of this study and to clarify the differences.

One of the major barriers to weight management when eating out was that restaurant food tastes good. Taste influences restaurant selection and food choice (9,11), and palatability is associated with higher calorie intake (27). One possible way to address this barrier is to encourage people to be mindful as they eat, savoring each bite, maximizing the pleasure of eating while reducing the amount of food needed to be satisfied (28). Another barrier was not wanting to waste food. This barrier may be linked to the need to get the best value, which is determined by cost and portion size (3). Combined with excessively large portions endemic in many restaurants, not wanting to waste food can lead to overconsumption (29). If restaurants made nutrition information available on menus, including the portion sizes served, customers could weigh the cost of consumption in terms of calories and fat. Other approaches would be to provide restaurants with incentives to offer smaller portions or provide take-home containers concurrent with the meal.

### Limitations

One limitation of the study was the use of retrospective, self-reported data that may not be as accurate as monitoring the actual frequencies of strategies used. Also, this sample had a high level of education. Since some of the strategies and barriers are linked to knowledge, findings would most likely be different in samples with more diverse educational backgrounds.

Additional caution should be used when considering the results of the multiple comparisons made without a Bonferroni adjustment; multiple comparisons increased the risk of type I error. The findings related to sex differences need to be replicated; however, with the Bonferroni adjustment, none of the findings would have been significant even if the differences noted were true. The sex difference findings should be viewed as trends that require further study.

### Conclusions

This study contributes to the weight management literature by describing how often women and men use different strategies for managing weight when eating out and by describing the barriers faced to managing weight when eating out. To address the obesity epidemic, we need a comprehensive understanding of what contributes to excess intake. Knowledge about how restaurant eating contributes to weight gain and how people can combat that weight gain is needed to prevent chronic disease. Further research on how the restaurant setting affects weight management is needed to develop interventions that can compensate for this obesogenic food environment.

## Figures and Tables

**Table 1:** Frequency of Using Strategies to Manage Weight When Eating in a Restaurant, by Sex

Strategy	Mean (SD) Score^b^	%^a^				

		Rarely	On Occasion	Sometimes	Often	Usually
**Avoid high-calorie/high-fat appetizers**						
Women	2.9 (1.3)	24	13	23	30	10
Men	2.6 (1.5)	33	24	13	13	18
**Have the bread/chip basket removed from table**						
Women	1.5 (0.8)	75	12	12	3	0
Men	1.3 (0.7)	78	13	7	2	0
**Share an appetizer^c^**						
Women	3.4 (1.4)	11	19	20	20	30
Men	2.8 (1.4)	22	24	20	20	14
**Substitute appetizer for a meal^c^**						
Women	2.5 (1.2)	26	23	28	18	6
Men	1.9 (1.1)	51	22	18	7	2
**Have a salad as a meal^c^**						
Women	2.9 (1.2)	20	18	24	31	7
Men	2.2 (1.2)	34	27	24	9	6
**Order salad dressing on the side**						
Women	3.0 (1.6)	31	12	18	11	29
Men	2.4 (1.7)	52	13	4	9	22
**Choose salad dressings lower in calories**						
Women	2.6 (1.5)	40	9	22	11	18
Men	2.6 (1.6)	36	18	14	11	20
**Have low-calorie snack before you go out to eat**						
Women	1.6 (0.8)	64	15	20	1	0
Men	1.9 (1.2)	58	11	22	4	6
**Instead of sugar-filled drinks, drink water, diet soft drinks, or unsweetened tea**						
Women	3.8 (1.5)	12	11	11	18	48
Men	4.0 (1.3)	6	11	18	6	60
**Have half of your meal boxed up to take home^c^**						
Women	3.0 (1.4)	18	21	28	15	19
Men	2.4 (1.3)	33	29	16	13	9
**Trim fat or skin from meats**						
Women	3.2 (1.6)	22	14	15	16	32
Men	3.1 (1.5)	23	9	26	17	24
**Share food in your meal with dining partner^c^**						
Women	3.0 (1.3)	18	13	33	22	14
Men	2.6 (1.2)	20	26	34	13	7
**Avoid deep-fried foods**						
Women	2.9 (1.4)	20	20	22	22	16
Men	3.0 (1.5)	20	24	18	13	26
**Choose steamed vegetables**						
Women	3.5 (1.3)	10	17	14	34	24
Men	3.4 (1.3)	7	18	27	20	27
**When eating soup, choose broth-based rather than cream-based**						
Women	2.8 (1.4)	25	22	25	7	21
Men	2.4 (1.4)	38	20	20	7	14
**Select foods that are baked, grilled, broiled, poached, or steamed**						
Women	3.5 (1.2)	4	17	27	25	26
Men	3.3 (1.2)	6	22	33	17	22
**Ask chef to prepare items in low-calorie or low-fat manner**						
Women	1.6 (0.9)	61	20	17	1	1
Men	1.6 (1.2)	73	11	7	2	7
**Eat bread without butter or oil**						
Women	2.4 (1.3)	33	22	22	12	10
Men	2.8 (1.5)	26	24	18	11	22
**Use salsa or other low-fat toppings for baked potatoes instead of sour cream, butter, cheese, or bacon**						
Women	2.4 (1.5)	41	16	18	12	13
Men	2.6 (1.5)	37	7	32	6	18
**Choose meals that have fruits and vegetables as main ingredients**						
Women	3.0 (1.4)	16	20	24	21	19
Men	2.8 (1.3)	22	20	27	18	13
**Choose foods made with whole grains**						
Women	3.2 (1.3)	10	24	24	19	23
Men	3.3 (1.4)	17	15	15	28	26
**Use mustard rather than mayonnaise, ketchup, or "special sauce" on sandwiches**						
Women	3.0 (1.6)	28	12	22	13	25
Men	3.0 (1.5)	26	14	16	24	20
**Eat fresh fruit for dessert**						
Women	3.0 (1.3)	16	15	32	20	16
Men	3.0 (1.4)	18	22	22	16	22
**Select low-fat, low-calorie, or sugar-free desserts**						
Women	2.3 (1.4)	38	23	16	11	11
Men	2.4 (1.4)	36	20	20	11	13
**Share a dessert or eat only a few bites**						
Women	3.0 (1.4)	19	16	31	13	21
Men	2.8 (1.4)	22	20	34	6	18
**Stop eating when full**						
Women	3.5 (1.2)	4	20	27	23	26
Men	3.6 (1.3)	6	18	13	32	32
**Reduce frequency of eating out**						
Women	2.8 (1.3)	18	21	34	12	14
Men	2.8 (1.4)	20	20	33	7	18
**Limit or avoid alcohol^d^**						
Women	4.1 (1.8)	11	11	19	10	14
Men	4.1 (1.7)	7	13	20	11	20
**Drink alcohol with meal instead of before meal^d^**						
Women	3.9 (2.0)	19	12	9	12	14
Men	3.8 (1.9)	16	14	16	11	13
**If you have alcoholic beverages, choose a low-calorie version^d^**						
Women	3.5 (2.1)	32	9	12	7	8
Men	3.2 (2.1)	33	16	16	2	4

a Percentages may not total 100 because of rounding.

b Scores ranged from 1 (rarely) to 5 (usually).

c Difference between men's and women's scores significant at *P* < .05.

d These questions were not applicable to 29% of men and 33%-35% of women (depending on the question).

**Table 2:** Magnitude of Barriers to Managing Weight When Eating in a Restaurant, by Sex

Barrier	Mean (SD) Score^b^	%^a^				

		Not a Barrier	Small Barrier	Moderate Barrier	Major Barrier	Overwhelming Barrier
**Difficult to have bread/chip basket removed from table^c^**						
Women	2.4 (1.3)	34	18	23	22	3
Men	1.9 (1.1)	51	20	18	9	2
**Lack of healthy menu items**						
Women	2.6 (1.2)	24	21	33	16	6
Men	2.6 (1.1)	20	24	38	14	4
**Healthy menu items more expensive**						
Women	2.4 (1.2)	33	15	34	12	6
Men	2.4 (1.2)	33	18	26	20	2
**Eating out is a special occasion, so I want to splurge**						
Women	2.3 (1.2)	36	21	26	13	4
Men	2.3 (1.1)	31	27	24	16	2
**Difficulty getting others to share an entrée or dessert**						
Women	1.7 (0.9)	50	31	17	1	1
Men	1.8 (0.9)	44	34	18	4	0
**Eating out when not hungry to please others**						
Women	1.9 (1.2)	53	20	15	6	7
Men	2.0 (1.1)	46	24	18	7	4
**Hard to ask the wait staff to box up meal to take home**						
Women	1.4 (0.8)	78	13	4	2	2
Men	1.3 (0.8)	82	13	0	4	2
**Wait staff not aware of what ingredients are in foods**						
Women	2.0 (1.1)	38	32	21	4	4
Men	2.0 (1.1)	46	27	13	14	0
**Lack knowledge about best menu choices at restaurants**						
Women	2.0 (1.1)	44	25	21	7	3
Men	2.1 (1.1)	41	24	24	7	4
**Uncomfortable asking to modify menu item preparation to lower calorie or fat content**						
Women	2.0 (1.2)	46	22	18	10	4
Men	2.2 (1.2)	43	20	22	9	6
**Busy lifestyle results in being overly hungry when eating out**						
Women	2.7 (1.4)	23	29	16	20	12
Men	2.5 (1.1)	22	24	33	20	0
**Unsure about appropriate portion sizes**						
Women	2.2 (1.3)	42	22	17	11	8
Men	2.0 (1.0)	36	33	22	7	2
**Restaurant food tastes good**						
Women	2.8 (1.4)	23	23	24	15	14
Men	2.8 (1.3)	24	13	31	26	7
**Unsure about calorie content of restaurant foods**						
Women	2.7 (1.4)	29	16	25	13	16
Men	2.4 (1.2)	29	29	26	11	6
**Don't like to waste food**						
Women	2.5 (1.4)	36	15	19	19	11
Men	2.8 (1.4)	27	14	27	14	16
**Don't like taking leftovers home**						
Women	1.6 (1.4)	70	12	11	2	4
Men	1.8 (1.1)	55	23	11	6	4
**Want to eat same things as those I'm dining with**						
Women	1.8 (1.1)	59	14	16	9	1
Men	1.8 (0.9)	47	33	16	4	0
**Feel deprived if cannot openly choose from menu**						
Women	2.0 (1.2)	52	21	12	10	6
Men	2.0 (1.0)	42	27	22	7	2
**Enjoy alcohol when going out to eat**						
Women	2.0 (1.3)	55	16	17	5	8
Men	1.8 (1.1)	58	17	19	2	4
**Important to get your money's worth when eating out**						
Women	2.3 (1.4)	42	16	21	12	9
Men	2.4 (1.3)	33	20	31	7	9
**Cannot take only a few bites of dessert**						
Women	2.4 (1.5)	41	18	18	8	16
Men	2.2 (1.1)	36	22	33	6	4
**Difficult to pay attention to my body's cues of fullness when eating out**						
Women	2.4 (1.4)	37	22	14	13	13
Men	2.2 (1.2)	33	29	26	7	6
**Limited time to eat out, so I need to choose fast food**						
Women	2.4 (1.4)	38	19	20	12	11
Men	2.1 (1.2)	47	13	24	14	2
**Lack of convenient restaurants in my area that have healthy menu items**						
Women	2.4 (1.5)	41	19	18	7	16
Men	2.2 (1.2)	40	24	22	11	4
**Social pressure to eat unhealthy menu items**						
Women	2.0 (1.3)	50	16	21	3	9
Men	1.7 (1.0)	60	14	20	6	0

a Percentages may not total 100 because of rounding.

b Scores ranged from 1 (not a barrier) to 5 (overwhelming barrier).

c Difference between men's and women's scores significant at *P* = .01.
